# Stimulation of Ca^2+^‐ATPase Transport Activity by a Small‐Molecule Drug

**DOI:** 10.1002/cmdc.202100350

**Published:** 2021-08-26

**Authors:** Giacomo Sordi, Andrea Goti, Howard S. Young, Ilaria Palchetti, Francesco Tadini‐Buoninsegni

**Affiliations:** ^1^ Department of Chemistry “Ugo Schiff” University of Florence Via della Lastruccia 3–13 50019 Sesto Fiorentino Italy; ^2^ Department of Biochemistry University of Alberta Edmonton, Alberta T6G 2H7 Canada; ^3^ Present address: PQE Group 50066 Reggello Florence Italy

**Keywords:** calcium translocation, drug-protein interactions, membrane transporter, reconstituted proteoliposomes, sarcoplasmic reticulum vesicles

## Abstract

The sarco(endo)plasmic reticulum Ca^2+^−ATPase (SERCA) hydrolyzes ATP to transport Ca^2+^ from the cytoplasm to the sarcoplasmic reticulum (SR) lumen, thereby inducing muscle relaxation. Dysfunctional SERCA has been related to various diseases. The identification of small‐molecule drugs that can activate SERCA may offer a therapeutic approach to treat pathologies connected with SERCA malfunction. Herein, we propose a method to study the mechanism of interaction between SERCA and novel SERCA activators, i. e. CDN1163, using a solid supported membrane (SSM) biosensing approach. Native SR vesicles or reconstituted proteoliposomes containing SERCA were adsorbed on the SSM and activated by ATP concentration jumps. We observed that CDN1163 reversibly interacts with SERCA and enhances ATP‐dependent Ca^2+^ translocation. The concentration dependence of the CDN1163 effect provided an EC_50_=6.0±0.3 μM. CDN1163 was shown to act directly on SERCA and to exert its stimulatory effect under physiological Ca^2+^ concentrations. These results suggest that CDN1163 interaction with SERCA can promote a protein conformational state that favors Ca^2+^ release into the SR lumen.

## Graphical abstract

**Electric insight**: The interaction between the small‐molecule drug CDN1163 and sarcoplasmic reticulum Ca^2+^−ATPase was investigated by electrical current measurements. It was shown that CDN1163 reversibly binds to the ATPase protein and significantly enhances ATP‐dependent calcium translocation. CDN1163 was able to exert its stimulatory effect on Ca^2+^−ATPase transport activity under physiological Ca^2+^ concentrations.

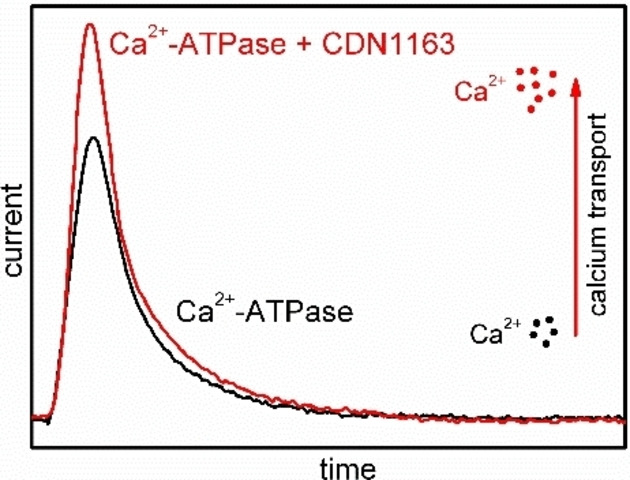

## Introduction

The sarco(endo)plasmic reticulum Ca^2+^−ATPase (SERCA), belonging to the superfamily of membrane transporters known as P‐type ATPases, is an intracellular membrane‐associated enzyme of approximately 110 kDa.[^1^, ^2^, ^3^] In muscle cells SERCA couples the hydrolysis of one ATP molecule to the transport of two Ca^2+^ ions against their electrochemical potential gradient from the cytoplasm into the lumen of the sarcoplasmic reticulum (SR). Ca^2+^ uptake in the SR lumen by SERCA plays an essential role in regulating cytoplasmic Ca^2+^ concentration, which is maintained at 0.1 μM; in this manner, SERCA induces muscle cell relaxation and contributes to intracellular Ca^2+^ homeostasis.

The SERCA transport cycle includes initial enzyme activation triggered by high affinity binding of two Ca^2+^ ions from the cytoplasmic side. This initial step is followed by enzyme phosphorylation by ATP and formation of a phosphorylated intermediate. A conformational transition of the phosphoenzyme favors translocation of Ca^2+^ ions across the membrane and their release into the SR lumen in exchange for two luminal protons. Hydrolytic cleavage of the phosphoenzyme (dephosphorylation) is followed by proton translocation and release to the cytosolic side, thus completing the transport cycle. Several crystal structures of SERCA in different conformational states in the transport cycle have been determined at atomic resolution, as reported in detailed reviews.[^1^, ^4^, ^5^, ^6^, ^7^, ^8^]

Given its role in controlling cellular Ca^2+^ homeostasis, dysfunction of the SERCA enzyme has been associated with pathological conditions and several diseases with a wide range of severity.[^3^, ^9^, ^10^] Impaired SERCA function leads to increased intracellular calcium concentration and triggers endoplasmic reticulum (ER) stress. ER stress is associated with a variety of common diseases, such as cardiovascular and neurodegenerative diseases, diabetes, obesity and metabolic disorders.[^11^, ^12^] Dysfunction of the SERCA pump also contributes to alteration in muscle contraction‐relaxation which can lead to various skeletal and cardiac muscle pathologies. It is therefore evident that SERCA represents an important target for the development of novel compounds with distinct therapeutic potential.

It has been proposed that pharmacological stimulation of SERCA activity may constitute an innovative and promising therapeutic approach to treat cardiovascular diseases, neurodegenerative diseases, i. e., Parkinson and Alzheimer disease, and diabetes.[^12^, ^13^] However, drugs that can stimulate an enzyme are rare. In fact, the task of activating an enzyme is much more demanding than inhibiting it. A few compounds have been identified that are able to stimulate SERCA activity. Such SERCA activators include the drug istaroxime that exerts a stimulatory effect on the cardiac isoform of SERCA (SERCA2a),^[14]^ the compounds CP‐154526 and Ro 41‐0960 that were also reported to activate the SERCA2a isoform,^[15]^ a pyridone derivative with potential therapeutic applications in heart failure,^[16]^ and a newly discovered regulatory peptide (dwarf open reading frame, DWORF) that enhances SERCA activity in muscle.^[17]^

**Figure 1:**
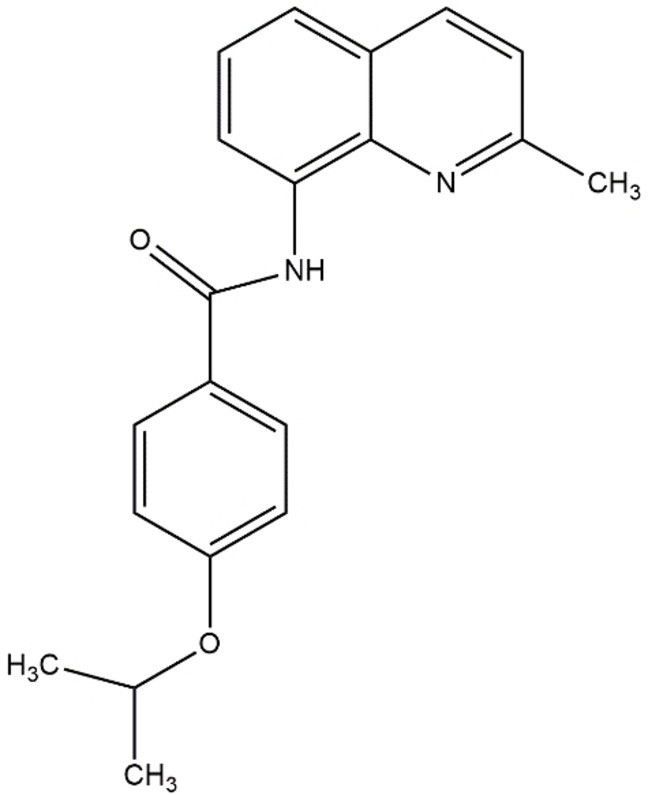
Chemical structure of CDN1163.

Recent studies reported the effects of the quinoline‐amide compound CDN1163 that has been proposed as a SERCA activator.[^18^, ^19^, ^20^, ^21^, ^22^, ^23^, ^24^] The compound CDN1163 (Figure 1) was selected by a high‐throughput screening of a large chemical library using a fluorescence resonance energy transfer (FRET) assay on SERCA labeled with fluorescent dyes in a reconstituted membrane system.^[18]^ CDN1163 displays favorable properties in terms of adsorption, distribution, metabolism and excretion, including metabolic and plasma stability, high permeability across biological membrane and lack of cardiotoxicity, i. e. hERG inhibition.^[21]^ It was suggested that CDN1163 binds to SERCA and enhances the Ca^2+^−ATPase activity. In particular, CDN1163 showed similar concentration dependence for FRET and ATPase activity assays, indicating a link between structural and functional effects on the SERCA enzyme.^[19]^ It is noteworthy that CDN1163 was tested in a selectivity screen to evaluate its effect on various ion pumps and channels and was found to be only active toward SERCA.^[18]^

**Figure 2:**
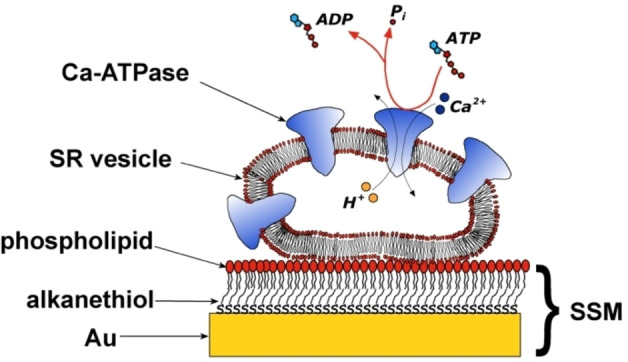
Schematic diagram of a SSM with an adsorbed SR vesicle containing SERCA (not drawn to scale).

In this study we have employed a biosensing approach based on a solid supported membrane (SSM) to characterize the mechanism of interaction between SERCA and CDN1163. This method has been used to investigate in detail the ion translocation mechanism of the SERCA enzyme.^[25]^ The SSM represents a convenient model system for a bilayer lipid membrane, and consists of a hybrid alkanethiol/phospholipid bilayer supported by a gold electrode (Figure 2).[^26^, ^27^] The hybrid bilayer is characterized by a high mechanical stability so that solutions can be rapidly exchange at the SSM surface.^[28]^

Native SR vesicles or reconstituted proteoliposomes containing SERCA were adsorbed on the SSM (Figure 2) and activated by ATP concentration jumps through the solution exchange technique. The observed current signals allow the measurement of charge (calcium ions) movement through the protein across the membrane. We found that calcium translocation by SERCA is significantly enhanced in the presence of CDN1163. A reversible and direct interaction between SERCA and CDN1163 was observed. Electrical measurements were performed to evaluate the concentration dependence of the CDN1163 stimulatory effect on SERCA transport activity.

## Results and Discussion

Drug/protein interactions can be conveniently monitored on a SSM. In particular, the SSM method has successfully been employed to evaluate the effects of pharmacologically relevant compounds on P‐type ATPases.^[29]^ The mechanisms of interaction of various inhibitory compounds with SERCA were investigated using the SSM method.^[13]^

The present research aims at investigating the stimulatory effect of the compound CDN1163 on the transport activity of the SERCA enzyme. The pharmacological properties of CDN1163 have been evaluated on different cell lines and animal models, providing a proof‐of‐concept that CDN1163 has the potential to become an effective therapeutic approach for diabetes and metabolic dysfunction,^[20]^ and muscle diseases.[^23^, ^24^] Herein, we employed the SSM method to characterize the interaction of CDN1163 with SERCA with the aim of elucidating at a molecular level the ability of CDN1163 to enhance ATP‐dependent translocation of Ca^2+^ ions by SERCA.

**Figure 3:**
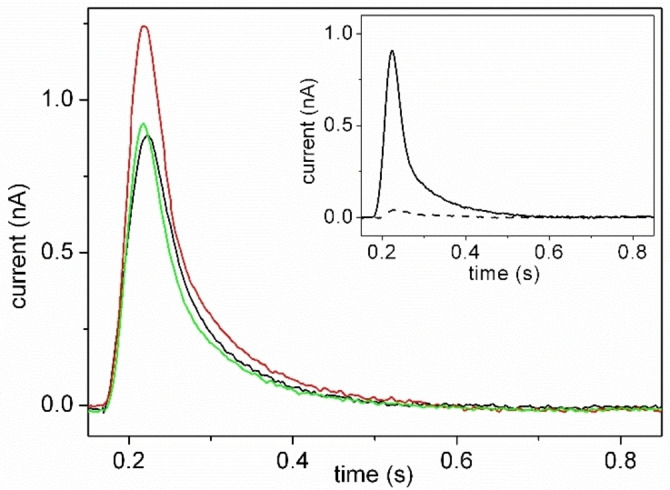
Current signals induced by 100 μM ATP concentration jumps on native SR vesicles in the presence of 10 μM free Ca^2+^ were measured in the absence of CDN1163 (initial control, black line), in the presence of 10 μM CDN1163 (red line), and again in the absence of CDN1163 by replacing the solution containing CDN1163 with one with no CDN1163 added (final control, green line). The inset shows current signals after 100 μM ATP concentration jumps in the absence (black solid line) and in the presence of 100 nM thapsigargin (black dashed line).

A convenient experimental system for functional studies of SERCA is provided by rabbit skeletal muscle SR vesicles, which contain a high amount of SERCA (SERCA1a isoform). We therefore performed current measurements on native SR vesicles that were adsorbed on a SSM electrode and subjected to ATP concentration jumps through fast solution exchange. A 100 μM ATP jump in the presence of 10 μM free Ca^2+^ induced a current signal (black line in Figure 3) that was fully suppressed (inset of Figure 3) by the potent and highly selective SERCA inhibitor thapsigargin,^[30]^ that binds irreversibly to the Ca^2+^‐free SERCA and stabilizes a catalytically inactive dead‐end complex with the ATPase.[^8^, ^31^] The inhibition experiment confirmed that the measured electrical current was related to SERCA activity. In particular, the observed current signal was attributed to an electrogenic event in the SERCA transport cycle corresponding to ATP‐dependent translocation and release of calcium ions into the SR vesicle interior.^[32]^ It is worth mentioning that a 100 μM ATP jump in the absence of Ca^2+^ ions yielded no electrical signal.^[32]^ We point out that the SSM method allows pre‐steady‐state measurements of charge movements within the first transport cycle of the ATPase, while steady‐state currents are not measured.^[32]^

Interestingly, if the SR vesicles adsorbed on the SSM were first incubated with a solution containing CDN1163 for 15 minutes and the ATP concentration jump was then performed in the presence of CDN1163, a significantly higher current signal was observed (red line in Figure 3). The translocated charge, which was obtained by integration of the current signal recorded at 10 μM CDN1163, was about 30 % higher than that measured in the absence of CDN1163 (initial control, black line in Figure 3). This result indicates that CDN1163 exerts a remarkable stimulatory effect on SERCA by enhancing ATP‐dependent calcium translocation. It is worth noting that a ∼30 % increase in SERCA2a ATPase activity at saturating [Ca^2+^] was obtained in the presence of 10 μM CDN1163, as previously reported.^[19]^

Finally, the solution containing CDN1163 was replaced in the flow‐through cuvette, which contained the SSM electrode, by a buffered solution with no CDN1163 added (see Experimental Section for details). After incubating the SR vesicles adsorbed on the SSM with the CDN1163‐free solution for 5 minutes the ATP jump was repeated in the absence of CDN1163 (final control, green line in Figure 3). We observed that the ATP‐induced current signal was almost completely restored to the level obtained initially (compare black and green lines in Figure 3), thus suggesting that the interaction of CDN1163 with SERCA is fully reversible.

It is worth noting that the time course of the current signals was not modified by the presence of CDN1163. The decay time constant of the signal measured in the presence of CDN1163 (48 ms, red line in Figure 3) was almost identical to that obtained in the absence of the compound (52 ms, black line in Figure 3). Such decay time constants were determined by fitting the current signal decay with a first‐order exponential decay function. As a general remark, the shape and time frame of the ATP‐induced electrical currents in the absence and presence of CDN1163 were similar to those reported in previous studies of the SERCA transport activity with the SSM method.[^13^, ^25^, ^32^] These observations suggest that the kinetics of Ca^2+^ translocation by SERCA was not affected by CDN1163. Therefore, the increase of translocated charge observed in the presence of CDN1163 may be explained by different mechanisms: 1) A tentative explanation is that CDN1163 enhances ATP binding and/or Ca^2+^ binding to SERCA. However, this explanation seems less likely since high saturating concentrations of ATP (100 μM) and free Ca^2+^ (10 μM) were employed in our experiments; 2) An alternative explanation is that in the presence of CDN1163 the conformation equilibrium of SERCA is shifted towards a protein conformational state that favors the release of Ca^2+^ ions into the SR lumen, thereby increasing the amount of the translocated charge. This latter explanation seems in our opinion more likely.

**Figure 4:**
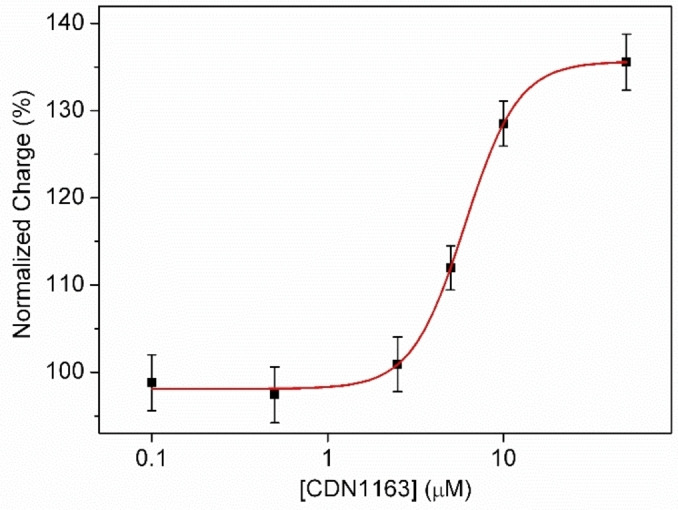
Normalized charges observed after 100 μM ATP concentration jumps on native SR vesicles in the presence of 10 μM free Ca^2+^ as a function of CDN1163 concentration. The charges were normalized with reference to the charge attained in the absence of CDN1163. The solid line represents the fitting curve to the ATP‐induced charges (EC_50_=6.0±0.3 μM). The error bars represent the standard error (SE) of three independent measurements.

To evaluate the concentration dependence of the CDN1163 effect, we recorded the electrical currents generated by SERCA following ATP concentration jumps in the presence of CDN1163 at increasing concentration. In these experiments, SERCA was incubated with CDN1163 for 15 minutes prior to the ATP jump, as mentioned above. Figure 4 shows the dependence of the translocated charge as a function of CDN1163 concentration. The measured charge was normalized with reference to the charge attained in the absence of CDN1163, taken as a control measurement. The Hill equation was then used to fit the normalized charges as shown in Figure 4. From this fitting a half‐maximum effective concentration (EC_50_) of 6.0±0.3 μM and a Hill coefficient (n_H_) of 2.9±0.3 were determined.

Measurements of the ATPase turnover rate (V_max_) at varying CDN1163 concentration on various SERCA isoforms (SERCA1a, SERCA2a and SERCA2b) also provided EC_50_ values in the micromolar range for CDN1163 and its structural analogs.[^18^, ^19^, ^20^] Thus, the EC_50_ value for CDN1163 indicates that this compound has a relatively high affinity for the SERCA enzyme.

Moreover, the n_H_ value >1 suggests that more than one CDN1163 molecule can interact with SERCA and binding of CDN1163 molecules to SERCA occurs with strong cooperativity. An alternative explanation is that CDN1163 binding to SERCA is conformation dependent and influences the conformational state of SERCA in a cooperative manner.

**Figure 5:**
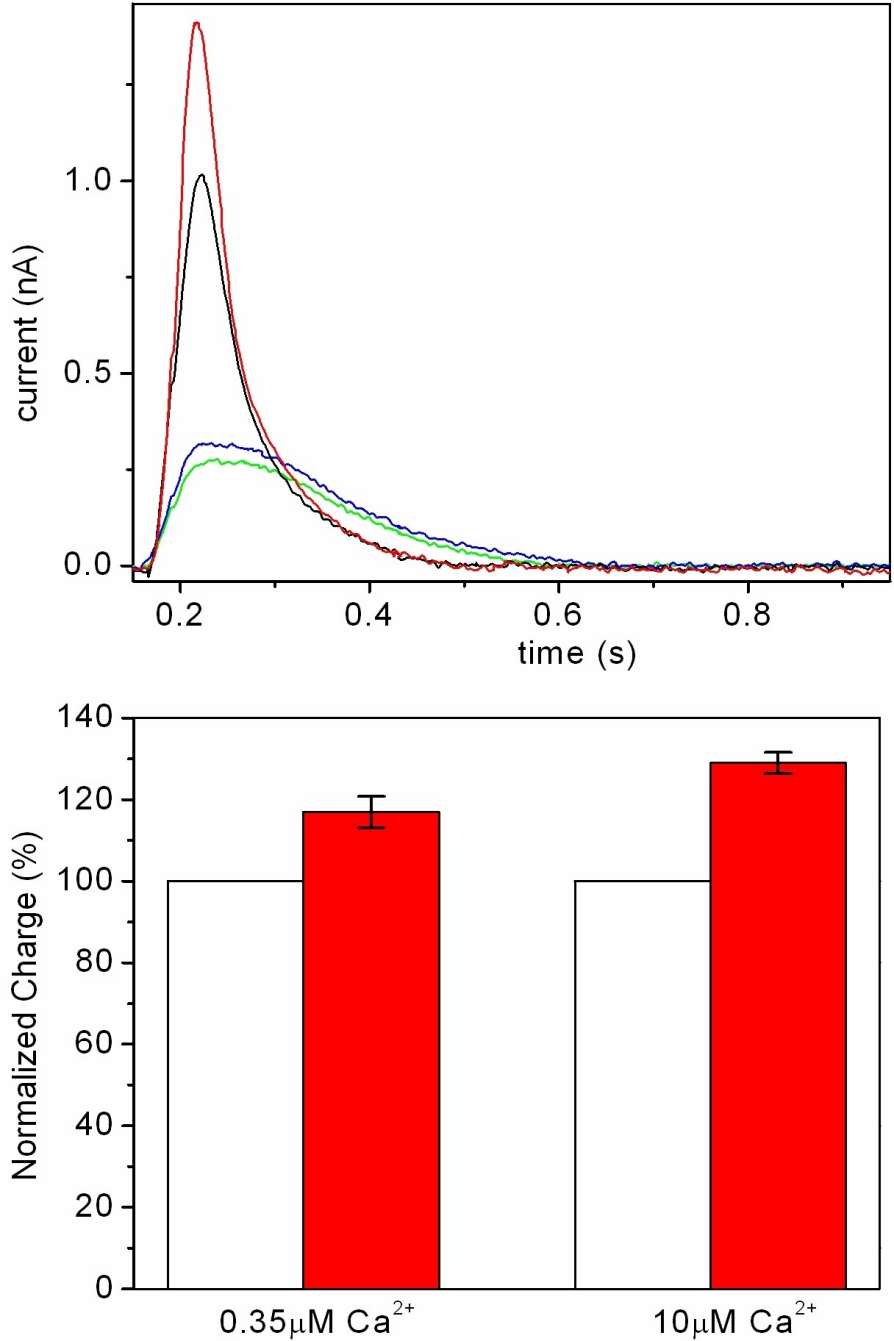
Current signals induced by 100 μM ATP concentration jumps on native SR vesicles (upper panel) in the presence of: 10 μM free Ca^2+^ (black line), 10 μM free Ca^2+^ and 10 μM CDN1163 (red line), 0.35 μM free Ca^2+^ (green line), 0.35 μM free Ca^2+^ and 10 μM CDN1163 (blue line). Normalized charges observed after 100 μM ATP concentration jumps on native SR vesicles (lower panel) in the presence of 0.35 μM and 10 μM free Ca^2+^ in the absence (white columns) and in the presence of 10 μM CDN1163 (red columns). The charges were normalized with reference to the charge attained in the absence of CDN1163. The error bars represent the standard error (SE) of three independent measurements.

In the ATP concentration jump experiments discussed above, a high saturating Ca^2+^ concentration (10 μM free Ca^2+^) was used that enabled full activation and maximal current response of the SERCA proteins adsorbed on the SSM. Previous studies also investigated the effect of CDN1163 on SERCA activity at high Ca^2+^ concentrations.[^18^, ^19^, ^20^, ^21^] However, it is known that the cytoplasmic Ca^2+^ concentration in the resting cell is very low, i. e. about 0.1 μM. We hence examined whether the stimulatory effect of CDN1163 on SERCA could still be observed at low calcium concentrations. Therefore, ATP concentration jumps were performed at 0.35 μM free Ca^2+^ in the absence and in the presence of 10 μM CDN1163 (after SERCA incubation with CDN1163 for 15 minutes), and the corresponding current signals were measured (upper panel of Figure 5). Free Ca^2+^ concentrations lower than 0.3 μM yielded current signals of small amplitude and low signal‐to‐noise ratio and therefore hardly detectable. At 0.35 μM free Ca^2+^ an increase in current amplitude was observed in the presence of CDN1163 (compare green and blue lines in the upper panel of Figure 5), even if such an increase was less pronounced than that observed at 10 μM free Ca^2+^ (compare black and red lines in the upper panel of Figure 5). In particular, 10 μM CDN1163 increased charge displacement by ∼17 % (red column) with respect to the control measurement (white column, absence of CDN1163) at 0.35 μM free Ca^2+^, as shown in the lower panel of Figure 5. These results support the concept that CDN1163 is able to stimulate SERCA transport activity under physiological Ca^2+^ concentrations within the cell. The CDN1163‐induced increase in charge displacement observed at a low [Ca^2+^] was, however, about half that measured at a high saturating Ca^2+^ concentrations (∼30 %, lower panel of Figure 5).

Finally, it is noteworthy that the time course of the current signals at 0.35 μM free Ca^2+^ was not significantly modified by the presence of CDN1163. In fact, almost identical decay time constants were determined for the signals measured in the absence (159 ms, green line in the upper panel of Figure 5) and in the presence of CDN1163 (150 ms, blue line in the upper panel of Figure 5), thereby confirming that CDN1163 did not affect the calcium translocation kinetics even at low calcium concentration. We point out that the slower current signal decay at 0.35 μM free Ca^2+^ with respect to that observed at 10 μM free Ca^2+^ is consistent with a slower turnover of the SERCA enzyme under low non‐saturating [Ca^2+^].

Previous investigations of the CDN1163 effects on skeletal muscle and cardiac SR vesicles suggested that CDN1163 acts directly on SERCA.^[18]^ It is worth mentioning that skeletal and cardiac SR preparations also contain SERCA regulatory transmembrane proteins, the most well‐known being phospholamban (cardiac muscle) and sarcolipin (skeletal muscle).

**Figure 6:**
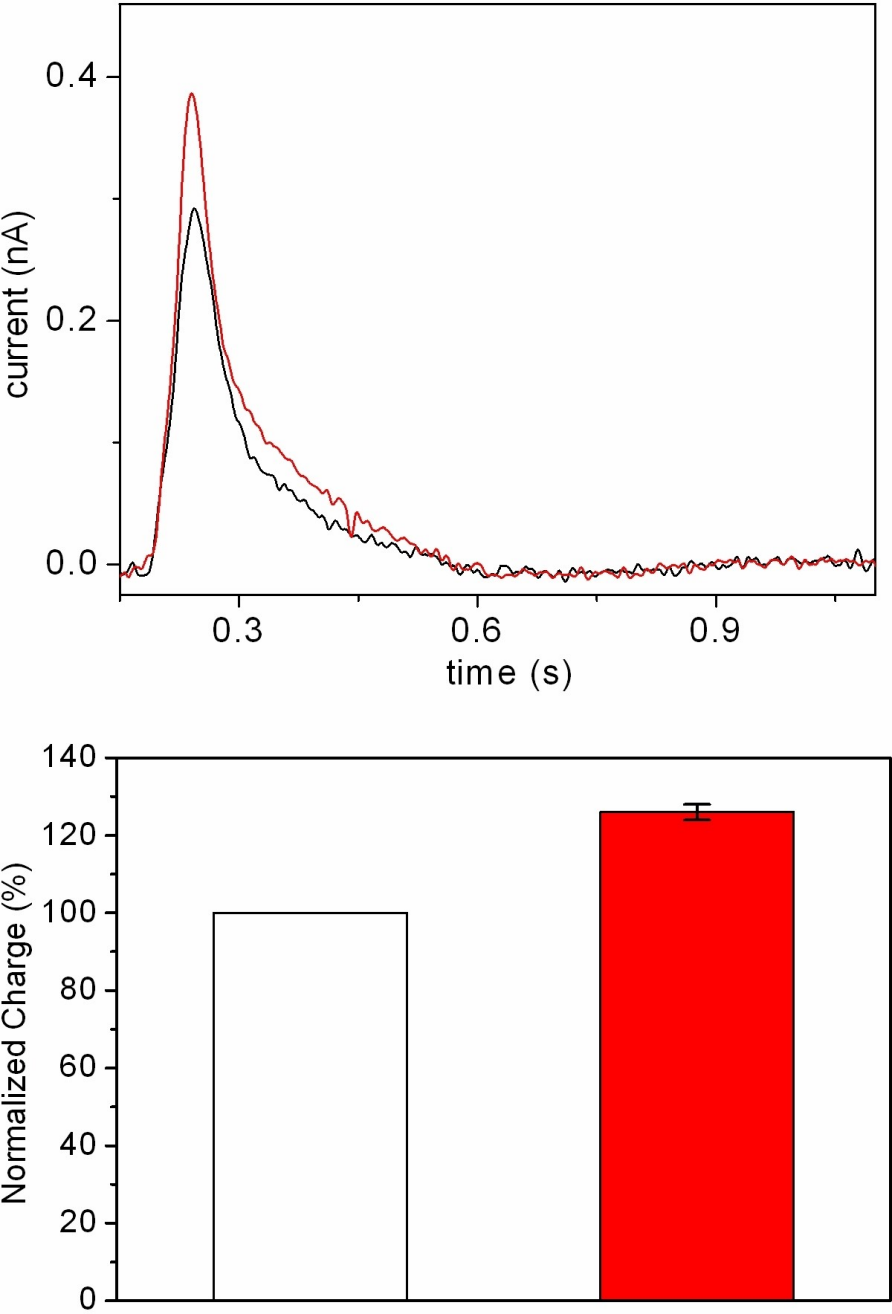
Current signals induced by 100 μM ATP concentration jumps on reconstituted proteoliposomes in the presence of 10 μM free Ca^2+^ (upper panel) were measured in the absence (black line) and in the presence (red line) of 10 μM CDN1163. Normalized charges observed after 100 μM ATP concentration jumps in the presence of 10 μM free Ca^2+^ (lower panel) in the absence (white column) and in the presence of 10 μM CDN1163 (red column). The charges were normalized with reference to the charge attained in the absence of CDN1163. The error bars represent the standard error (SE) of three independent measurements.

To find out whether CDN1163 directly interacts with the SERCA enzyme, we employed a simplified model membrane, consisting of reconstituted proteoliposomes of well‐defined lipid composition that only contain purified SERCA (SERCA1a isoform). Such proteoliposomes represent an effective mimic of native SR vesicles, and have been used extensively to characterize SERCA function and regulation.[^33^, ^34^, ^35^, ^36^, ^37^] Following adsorption on the SSM, the reconstituted proteoliposomes were activated by a 100 μM ATP concentration jump and the ATP‐induced current signal was detected (Figure 6). The proteoliposomes were then incubated with 10 μM CDN1163 for 15 minutes and the ATP jump was repeated in the presence of CDN1163. As shown in the upper panel of Figure 6, CDN1163 significantly increased the current amplitude (compare red and black lines), thereby confirming the stimulatory effect of CDN1163 on ATP‐dependent Ca^2+^ translocation by SERCA. It is noteworthy that the CDN1163‐induced increase in charge displacement (∼26 %, red column in Figure 6) observed with reconstituted proteoliposomes was almost identical to that obtained with native SR vesicles for the same CDN1163 concentration (lower panel of Figure 5). This result indicates that CDN1163 directly interacts with SERCA, and its stimulatory effect does not depend on the presence of SERCA regulatory partners such as sarcolipin that is found in the native SR vesicles.

## Conclusion

In this study we performed SSM‐based electrical measurements on skeletal muscle SR vesicles to investigate the interaction of SERCA with the compound CDN1163 that has been proposed as an effective SERCA activator. It was shown that CDN1163 reversibly interacts with SERCA and significantly enhances ATP‐dependent calcium translocation by SERCA under high and low (physiologically similar) Ca^2+^ concentrations. Our results indicate a relatively high affinity of CDN1163 for SERCA, and suggest that binding of CDN1163 to SERCA occurs in a cooperative manner. We propose that the binding site(s) for CDN1163 is localized in the transmembrane region of the ATPase, considering the lipophilic nature of CDN1163. We speculate that CDN1163 interaction with the SERCA transmembrane domain can promote a protein conformational state that favors the release of Ca^2+^ ions into the SR lumen, thereby enhancing calcium translocation.

The stimulatory effect of CDN1163 on ATP‐dependent Ca^2+^ translocation by SERCA was also observed with reconstituted proteoliposomes that only contained the purified SERCA1a isoform, thus indicating a direct interaction of CDN1163 with SERCA as previously suggested.

It is noteworthy that CDN1163 can exert a stimulatory effect on different SERCA isoforms (SERCA1a, SERCA2a, SERCA2b) indicating that CDN1163 is not isoform specific.^[21]^ Stimulation of SERCA transport activity by a small‐molecule drug, such as CDN1163, may represent an innovative therapeutic approach to treat various diseases that are associated with dysfunction of different SERCA isoforms, such as heart failure (SERCA2a), muscular dystrophy (SERCA1a), neurodegenerative disorders and diabetes (SERCA2b).

## Experimental Section

### Preparation of native SR vesicles and reconstituted proteoliposomes containing Ca^2+^−ATPase

Native SR vesicles were obtained by isolation from the fast twitch hind leg muscle of New Zealand white rabbit, as described.^[38]^ Protein concentration was determined by the Lowry method using bovine serum albumin as a standard.^[39]^ The total protein content of SR vesicles was 8.4 mg/mL. SERCA (isoform 1a) accounts for approximately 50 % of the microsomal protein.^[40]^

Following an established procedure, rabbit skeletal muscle SERCA (SERCA1a) was purified from SR vesicles by affinity chromatography.^[41]^ SERCA reconstitution in proteoliposomes containing egg yolk phosphatidylcholine and egg yolk phosphatidic acid has been described in detail.[^33^, ^34^, ^42^] The reconstituted proteoliposomes typically yield a lipid‐to‐protein molar ratio of approximately 120‐to‐1.^[33]^

### Electrical measurements

The SSM consists of an octadecanethiol monolayer covalently bound to the gold surface via the sulphur atom, with a diphytanoylphosphatidylcholine monolayer on the top of it (Figure 2).[^26^, ^27^] The SSM was mounted in a Plexiglas flow‐through cuvette and acted as the working electrode. The cuvette also contained a reference electrode, e. g. Ag/AgCl electrode, which was separated from the main fluid pathway by a salt bridge.[^43^, ^44^, ^45^] The cuvette and the entire fluid pathway were enclosed in a Faraday cage.

As a rule, two hours after forming the SSM the capacitance and resistance of the SSM attained constant values of the order of 0.2–0.4 μFcm^−2^ and 10–20 MΩcm^2^, respectively,^[28]^ in good agreement with previously reported electrochemical impedance spectroscopy (EIS) measurements.[^46^, ^47^]

Following SSM formation, native SR vesicles or reconstituted proteoliposomes incorporating SERCA were adsorbed on the SSM during an incubation time of 60 min. Electrical measurements indicate that the SSM capacitance and resistance remained practically constant after vesicle adsorption and throughout the time period of the experiment. This result is consistent with the adsorption of mainly intact vesicles on the SSM surface, as reported in a previous characterization of the vesicle adsorption process onto the SSM by employing surface plasmon resonance, EIS and atomic force microscopy.^[47]^

After vesicle adsorption on the SSM, SERCA was activated by a concentration jump of a suitable substrate, i. e. ATP (Figure 2), which was realized by rapidly changing from a non‐activating solution (without ATP, as specified below) to an activating solution, which contains ATP, at the SSM surface.[^28^, ^43^, ^48^] If the ATP concentration jump induced charge displacement across the membrane by the ATPase, an electrical current was detected. The transient nature of the observed electrical current is a consequence of the capacitively‐coupled system formed by the SSM and the vesicles adsorbed on it.[^43^, ^48^] The numerically integrated current transient is related to a net charge movement within the protein, which depends upon the electrogenic calcium translocation by SERCA. In particular, the electrical response of the transport protein can be monitored under potentiostatic conditions. In this case, movement of a net charge across the activated protein is compensated by a flow of electrons along the external circuit toward the gold electrode surface in order to keep constant the potential difference applied across the whole metal solution interphase.[^25^, ^32^, ^48^] This flow of electrons corresponds to the measured capacitive current, which is correlated with the protein‐generated current and is recorded as transient current signal.[^25^, ^43^, ^48^] Quite often, experiments are carried out under short‐circuit conditions, i. e. at zero applied voltage relative to the reference electrode.^[48]^

In ATP concentration jump experiments two buffered solutions were employed, the non‐activating and the activating solutions. The non‐activating solution contained 100 mM KCl, 25 mM MOPS (pH 7.0), 0.25 mM EGTA, 1 mM MgCl_2_, 0.25 mM CaCl_2_ (10 μM free Ca^2+^) or 0.12 mM CaCl_2_ (0.35 μM free Ca^2+^), and 1 mM DTT; the activating solution contained, in addition to the previously mentioned species, also 100 μM ATP. The free Ca^2+^ concentration was calculated with the computer program WinMAXC.^[49]^ 1 μM of the calcium ionophore A23187 (calcimycin) was used to prevent Ca^2+^ accumulation into the vesicles.

To evaluate the effect of CDN1163 (Sigma‐Aldrich) on the electrical current generated by SERCA, CDN1163 was added to both the non‐activating and the activating solutions. The vesicles adsorbed on the SSM were first incubated with the non‐activating solution containing CDN1163 for 15 min. Then the non‐activating solution was replaced with the activating solution containing CDN1163 and the ATP‐induced current signal was measured. The current signal observed in the presence of CDN1163 was compared with the electrical current obtained in the absence of CDN1163 (initial control measurement). To perform the final control experiment, the non‐activating and activating solutions containing CDN1163 were replaced in the experimental set‐up by the corresponding solutions with no CDN1163 added. The flow‐through cuvette with the SSM electrode was rinsed with the non‐activating solution without CD1163 to completely remove CDN1163 from the main fluid pathway. After incubating the vesicles adsorbed on the SSM with the CDN1163‐free solution for 5 minutes the ATP concentration jump was repeated in the absence of CDN1163 and the ATP‐induced current signals corresponding to the initial and final control measurements were compared.

The ATP concentration jump experiments were performed by employing the SURFE^2^R^One^ device (Nanion Technologies, Munich, Germany). The SSM sensor, the experimental setup, as well as the solution exchange technique have been described in detail.[^44^, ^45^, ^50^] The temperature was maintained at 22–23 °C for all the experiments.

To verify the reproducibility of the current transients on the same SSM, each single measurement was repeated 6 times and then averaged to improve the signal to noise ratio. Standard deviations did not exceed 5 %. Moreover, each set of measurements was usually reproduced using 3 different SSM sensors.

## Conflict of interest

The authors declare no conflict of interest.
